# Correction: Zhitkevich et al. HIV-1 Reverse Transcriptase Expression in HPV16-Infected Epidermoid Carcinoma Cells Alters E6 Expression and Cellular Metabolism, and Induces a Hybrid Epithelial/Mesenchymal Cell Phenotype. *Viruses* 2024, *16*, 193

**DOI:** 10.3390/v16040589

**Published:** 2024-04-11

**Authors:** Alla Zhitkevich, Ekaterina Bayurova, Darya Avdoshina, Natalia Zakirova, Galina Frolova, Sona Chowdhury, Alexander Ivanov, Ilya Gordeychuk, Joel M. Palefsky, Maria Isaguliants

**Affiliations:** 1 Chumakov Federal Scientific Center for Research and Development of Immune-and-Biological Products of Russian Academy of Sciences, 119991 Moscow, Russia; bayurova_eo@chumakovs.su (E.B.); avdoshina_dv@chumakovs.su (D.A.); frolova_ga@chumakovs.su (G.F.); gordeychuk_iv@chumakovs.su (I.G.); 2 Gamaleya National Research Center for Epidemiology and Microbiology, 123098 Moscow, Russia; aivanov@yandex.ru; 3 Centre for Precision Genome Editing and Genetic Technologies for Biomedicine, Engelhardt Institute of Molecular Biology, 119991 Moscow, Russia; nat_zakirova@mail.ru; 4 Division of Infectious Diseases, Department of Medicine, University of California, San Francisco, CA 94143, USA; sona.chowdhury@ucsf.edu (S.C.); joel.palefsky@ucsf.edu (J.M.P.); 5 Department of Microbiology, Tumor and Cell Biology, Karolinska Institutet, 171 77 Stockholm, Sweden

**Deletion of an Author**

Author Juris Jansons requested his exclusion from the authors of the original publication [1], alongside his affiliation “^4^ Biomedical Research and Study Centers, LV-1067 Riga”, as the given organization was against the participation of its employees in studies performed in part by researchers affiliated to Russian scientific organizations.

**Deletion of Affiliations**

Due to the same reasons, Maria Isaguliants requested the removal of her affiliation “^5^ Institute of Microbiology and Virology, Riga Stradins University, LV-1007 Riga, Latvia” and the replacement of her e-mail address with “maria.issagouliantis@ki.se”.

**Corrected Author Contributions Statement**

The original author contributions statement needs to be revised due to the deletion of Juris Jansons from the authors list. The new author contributions statement reads as follows: 

“A.Z. and E.B.—conceived the study, acquired the data, analyzed the collected material, wrote the manuscript; D.A., N.Z., G.F., and A.I.—acquired the data, analyzed the collected material; I.G.—acquired the data, discussed the results, secured funding; S.C.—discussed the results, reviewed the manuscript; J.M.P.—generated the concept, planned the experiment, discussed the results, secured funding, discussed the results, reviewed the manuscript; M.I.—generated the concept, planned the experiment, secured funding, discussed the results, wrote and reviewed the manuscript. All authors have read and agreed to the published version of the manuscript.”

**Corrected Funding**

The original publication included the funder “Latvian Science Fund, LZP 2021/1-0484” to “Maria Isaguliants, Juris Jansons”. This funder needs to be excluded. The corrected acknowledgements to funding should read: 

Twinning on DNA-Based Cancer Vaccines (VACTRAIN) project no.692293; Initiation Project INNOVIMMUNE, Swedish Institute 2017–2018 to M.I., and RFBR 17-54-30002 and R01 CA217715/CA/NCI NIH HHS/United States to J.M.P. and M.I. Metabolic analysis was performed from 2019 to 2020 with the support of the Ministry of Science and Higher Education of the Russian Federation (agreement 075-15-2019-1660).

The authors state that the scientific conclusions are unaffected. This correction was approved by the Academic Editor. The original publication has also been updated. 
