# Supplementary material for: HIV-1 Reverse Transcriptase Expression in HPV16-Infected Epidermoid Carcinoma Cells Alters E6 Expression and Cellular Metabolism, and Induces a Hybrid Epithelial/Mesenchymal Cell Phenotype
Source: Viruses. 2024 Jan 26;16(2):193. doi: 10.3390/v16020193 (PMC10892743; doi:10.3390/v16020193)
Supplement: Supplementary file 1 [file viruses-16-00193-s001.zip › viruses-2811363-supplementary.pdf]

SUPPLEMENTARY MATERIALS

Supplementary Table S1. List of primers used in the experiments. Fw – forward, rev – reverse.

Target sequence (gene)	Direction	Primer sequence
GAPDH	fw	5'-CAACGGATTTGGTCGTATTGG-3'
	rev	5'-GCAACAATATCCACTTTACCAGAGTTAA-3'
GUSB	fw	5'-CGTGGTTGGAGAGCTCATTTGGAA-3'
	rev	5'-ATTCCCCAGCACTCTCGTCGGT-3'
Reverse transcriptase of HIV-1 FSU_A strain (RT_A)	fw	5'-CTGGAGCTTGCTGAGAATAGAG-3'
	rev	5'-CACTGGTCCTGTCCTTGTTT-3'
E6FL	fw	5'-AAGTTAACCACAGTTATGC-3'
	rev	5'-TGTTCTAATGTTGTTCCAT-3'
*E6*I*	fw	5'-AGGAGCGACCCAGAAAGTTA-3'
	rev	5'-GCTTTTGACAGTTAATACACCTCAC-3'
*E6*II*	fw	5'-AGGAGCGACCCAGAAAGTTA-3'
	rev	5'-TACGTGTTCTTATGATCTCAGGTC-3'
E7	fw	5'-CGGACAGAGCCCATTACAATA-3'
	rev	5'-GAATGTCTACGTGTGTGCTTTG-3'
NRF2	fw	5'-TACTCCCAGGTTGCCCACA-3'
	rev	5'-CATCTACAAACGGGAATGTCTGC-3'
GCLC	fw	5'-GGATTTGGAAATGGGCAATTG-3'
	rev	5'-CTCAGATATACTGCAGGCTTGGAA-3'
NQO1	fw	5'-CCGTGGATCCCTTGCAGAGA-3'
	rev	5'-AGGACCCTTCCGGAGTAAGA-3'
A-TUBULIN	fw	5'-CCACAGTCATTGATGAAGTTCG-3'
	rev	5'-GCTGTGGAAAACCAAGAAGC-3'
Y-TUBULIN	fw	5'-CCCTCATCTGCCTTACTGGTTG-3'
	rev	5'-AGGTCCCTGATCTGTGCTCTGA-3'
E-CADHERIN	fw	5'-GAGTGCCAACTGGACCATTC-3'
	rev	5'-ACCCACCTCTAAGCCATCT-3'
N-CADHERIN	fw	5'-TGGAACGCAGTGTACAGAATCAG-3'
	rev	5'-TTGACTGAGGCGGGTGCTGAATT-3'
VIMENTIN	fw	5'-AGATGGCCCTTGACATTGAG-3'
	rev	5'-CCAGAGGGAGTGAATCCAGA-3'
TWIST1	fw	5'-GCAAGAAGTCGAGCGAAGAT-3'
	rev	5'-GCTCTGCAGCTCCTCGAA-3'
SNAI1	fw	5'-CGAAAGGCCTTCAACTGCAAAT-3'
	rev	5'-ACTGGTACTTCTTGACATCTG-3'
SNAI2	fw	5'-CTGGGCTGGCCAAACATAAG-3'
	rev	5'-CCTTGTCACAGTATTTACAGCTGAAAG-3'

Primers and probe specific to the 5'-end of the provirus genome: GAG	fw	5'-GGAGCTAGAACGATTGCAGTTA-3'
	rev	5'-GGTTGTAGCTGTCCCAGTATTTGTC-3'
	probe	5'-(FAM)-ACAGCCTTCTGATGTTTCTAACAGGCCAGG-(BHQ1)-3'
HB2	fw	5'-TCCGTGTGGATCGGCGGCTCCA-3'
	rev	5'-CTGCTTGCTGATCCACATCTG-3'
	probe	5'-(HEX)-CCTGGCCTCGCTGTCCACCTTCCA-(BHQ2)- 3'
Lentiviral insertion (PGK-seq/dir)	fw	5'-GGTGTTCCGCATTCTGCAAG-3'
Lentiviral insertion (pLVTseq200R)	rev	5'-GACAACGGGCCACAACTCC-3'

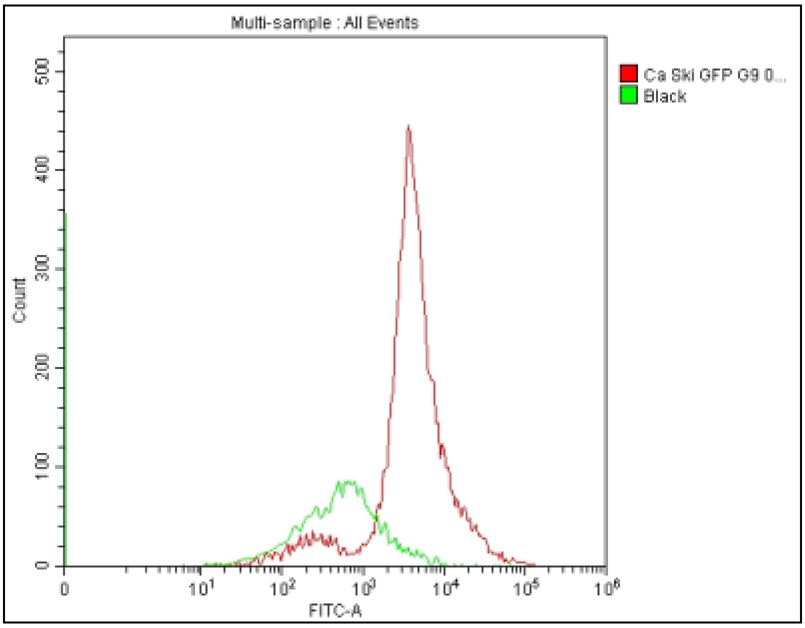

Supplementary Figure S1. Confirmation of GFP expression in Ca Ski subclone by flow cytometry. Histogram of events count recorded in FITC-A channel. Ca Ski GFP subclone (red) and control Ca Ski cell line (green). Events were counted among all events without gaiting.

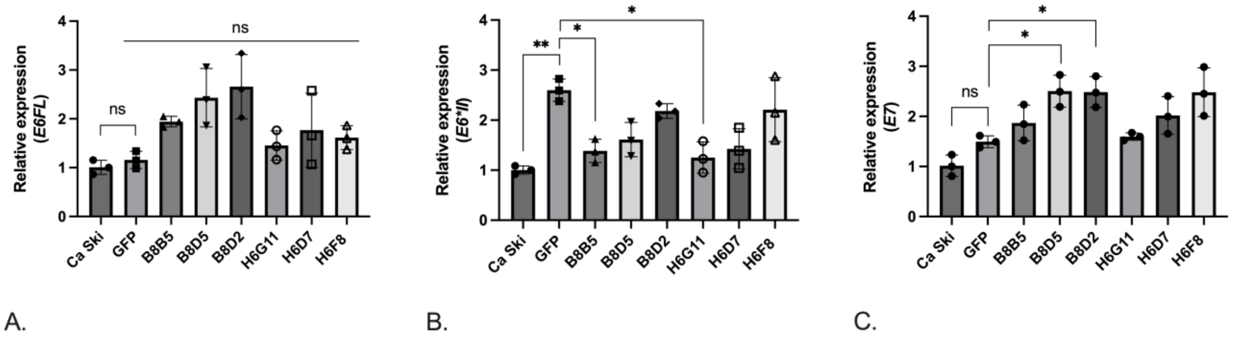

Supplementary Figure S2. Relative mRNA expression levels of *E6FL* (A), *E6*II* isoforms (B) and *E7* (C) HPV16 in Ca Ski subclones expressing consensus HIV-1 FSU_A reverse transcriptase compared Ca Ski expressing GFP and parental Ca Ski cells. Expression level was normalized to *GAPDH* expression and calculated as fold change compared to the parental Ca Ski line. Results are presented as mean ± SD, ns: not significant; ∗p < 0.05; ∗∗p < 0.01; ∗∗∗p < 0.001; ∗∗∗∗p < 0.0001 by Unpaired t test with Bonferroni correction.

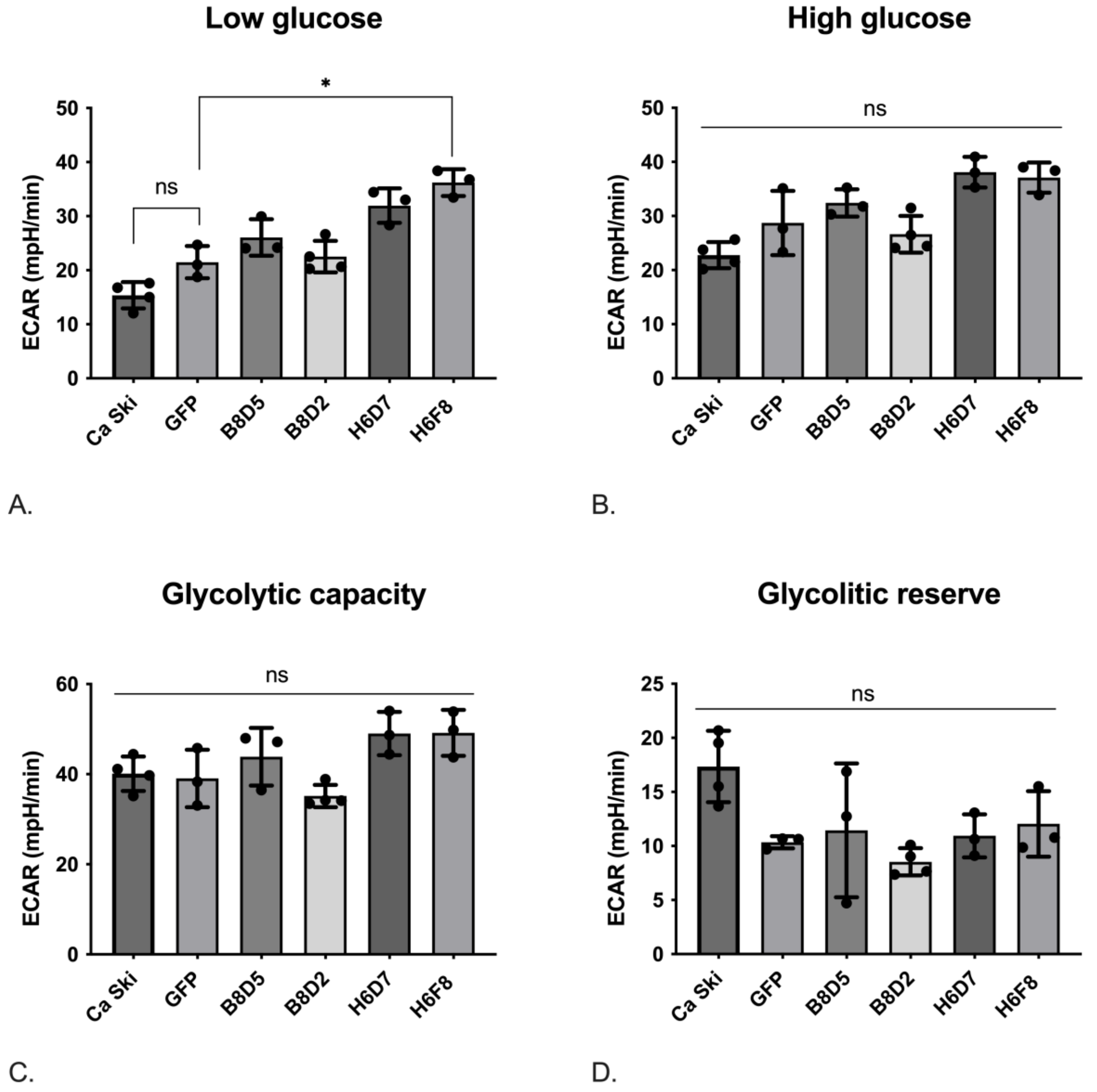

Supplementary Figure S3. The efficiency of glycolysis in Ca Ski subclones expressing HIV-1 reverse transcriptase (RT_A). Glycolysis efficiency of Ca Ski derivatives was evaluated using a Seahorse analyzer according to the Glycostress test method. Determination of ECAR in medium with standard low, 11 mM (A), and high, 30 mM glucose concentration (B); Maximum ECAR values upon stimulation with 1 µM oligomycin (C); The difference between the maximum glycolytic capacity and the level of glycolysis at 30 mM glucose (D). ECAR values are expressed in units of mpH/min and normalized to 1 mg of total cellular protein (mpH/min/Norm. Unit). Histograms are presented as mean ± SD for the analysis performed in quadruplicate. Significant difference was assessed using Unpaired t test with Bonferroni correction. ns: not significant; $*p < 0.05$; $**p < 0.01$; $***p < 0.001$; $****p < 0.0001$.

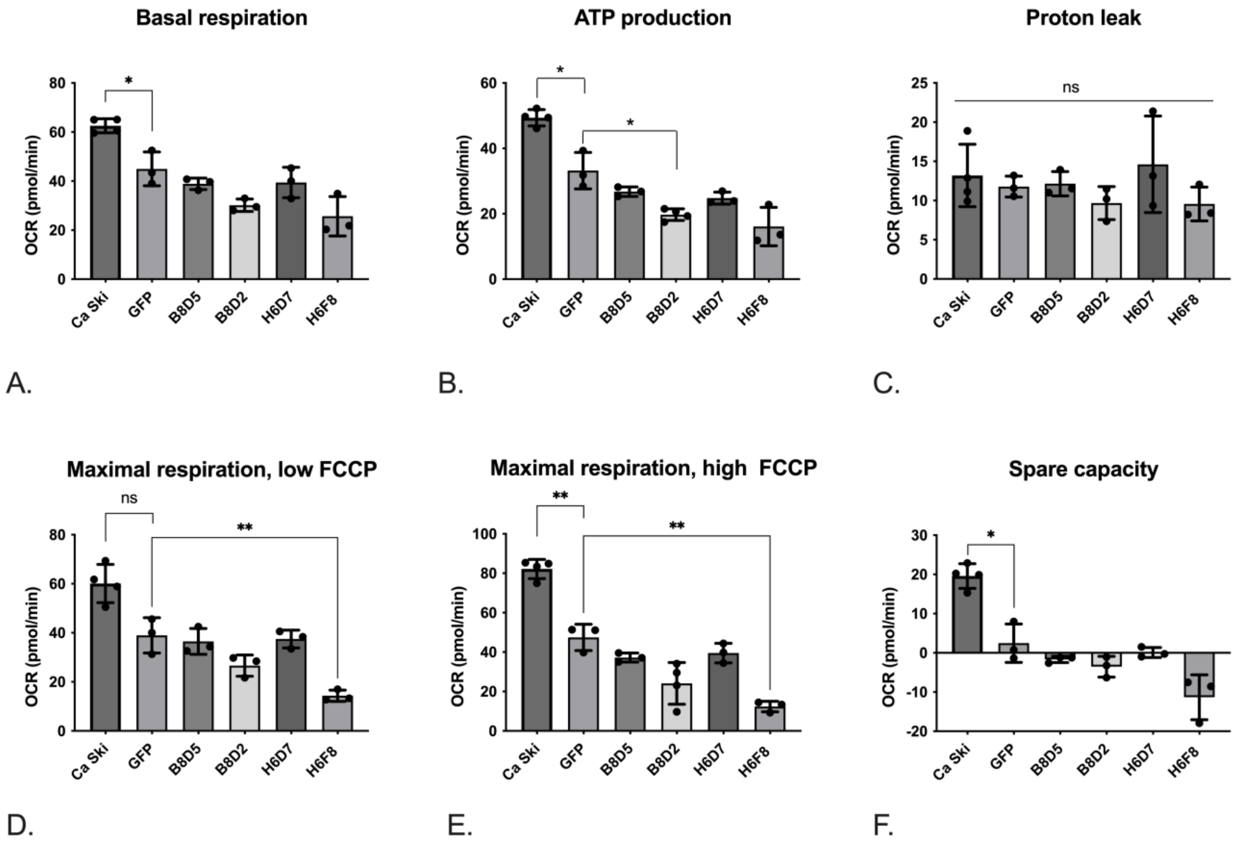

Supplementary Figure S4. The efficiency of the respiratory activity in Ca Ski subclones expressing HIV-1 reverse transcriptase (RT_A). Respiratory activity of Ca Ski derivatives was analyzed using Seahorse technology and the Mitostress reagent kit. Basal respiration rate determined as the difference between baseline and non-mitochondrial OCR values (A). ATP-bound OCR determined as the difference between the basal OCR and OCR inhibited by antimycin A (B); Maximum OCR value stimulated by the addition of FCCP at concentrations of 0.75 (C) and 1.5 µM (D); The difference between the levels of basal and maximum respiration (E). OCR values are expressed in units of pmol/min and normalized to 1 mg of total cellular protein (pmol/min/Norm. Unit). Histograms are presented as mean ± SD for the analysis performed in quadruplicate. Significant difference was assessed using Unpaired t test with Bonferroni correction. ns: not significant; $*p < 0.05$; $**p < 0.01$; $***p < 0.001$; $****p < 0.0001$.

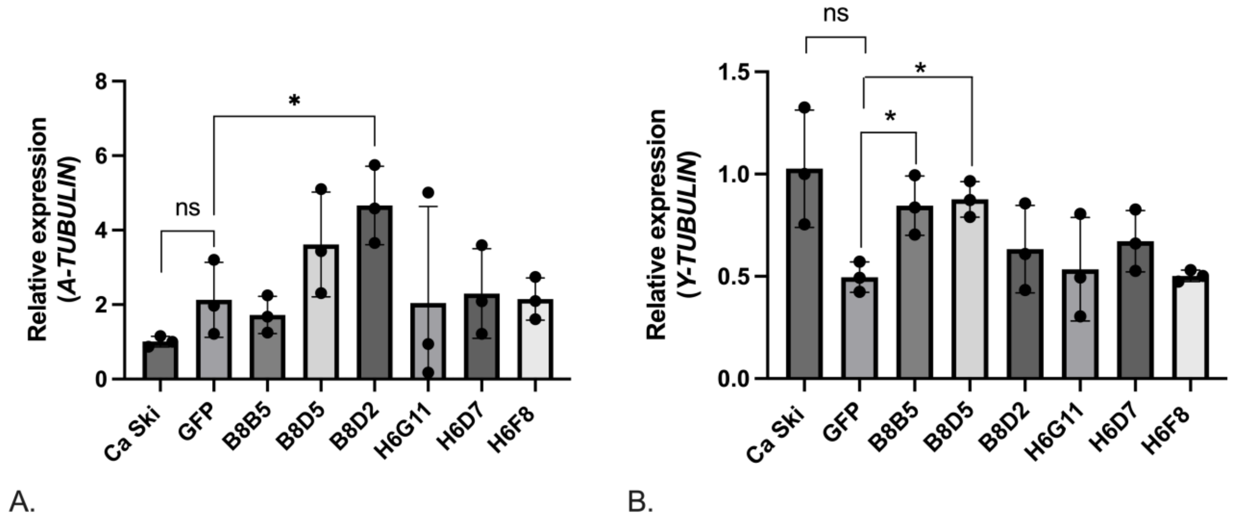

Supplementary Figure S5. Relative mRNA expression levels of *A-TUBULIN* (A) and *Y-TUBULIN* (B) in the derivatives of Ca Ski cells expressing variants of consensus HIV-1 FSU_A reverse transcriptase. Expression level was normalized to *GUSB* expression and calculated as fold change compared to the parental Ca Ski line. Results are presented as mean ± SD, ns: not significant; ∗p < 0.05; ∗∗p < 0.01; ∗∗∗p < 0.001; ∗∗∗∗p < 0.0001 by Unpaired t test with Bonferroni correction.

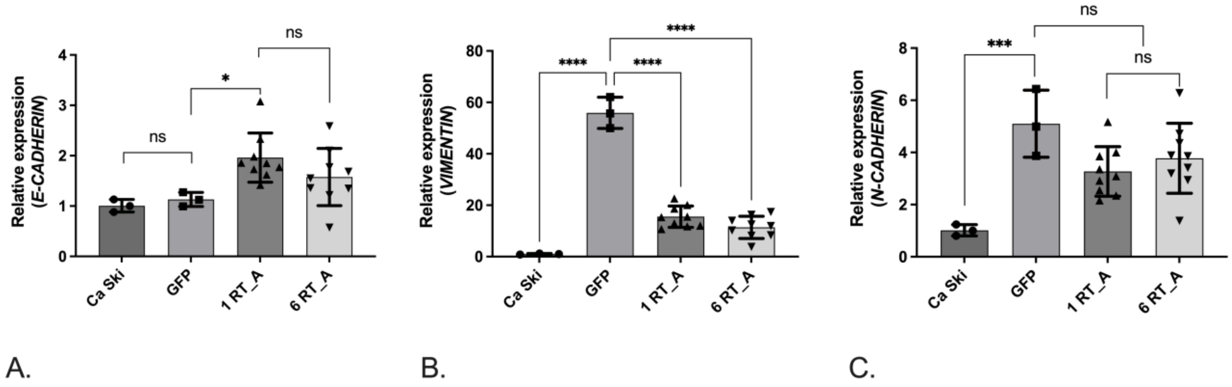

Supplementary Figure S6. Relative mRNA expression levels of *E-CADHERIN* (a), *VIMENTIN* (b), *N-CADHERIN* (c) in the derivatives of Ca Ski cells expressing HIV-1 RT_A. Ca Ski subclones with one genomic insert of RT_A coding sequence (B8B5, B8D5, B8D2) are designated as “1 RT_A”, and with six inserts as “6 RT_A” (H6G11, H6D7, H6F8), Ca Ski with six genomic inserts of GFP is given as a control. Expression level was normalized to *GAPDH* expression and calculated as fold change compared to the parental Ca Ski cell line. Results are presented as mean ± SD, ns: not significant; ∗p < 0.05; ∗∗p < 0.01; ∗∗∗p < 0.001∗∗∗∗p < 0.0001 by Unpaired t test with Bonferroni correction.

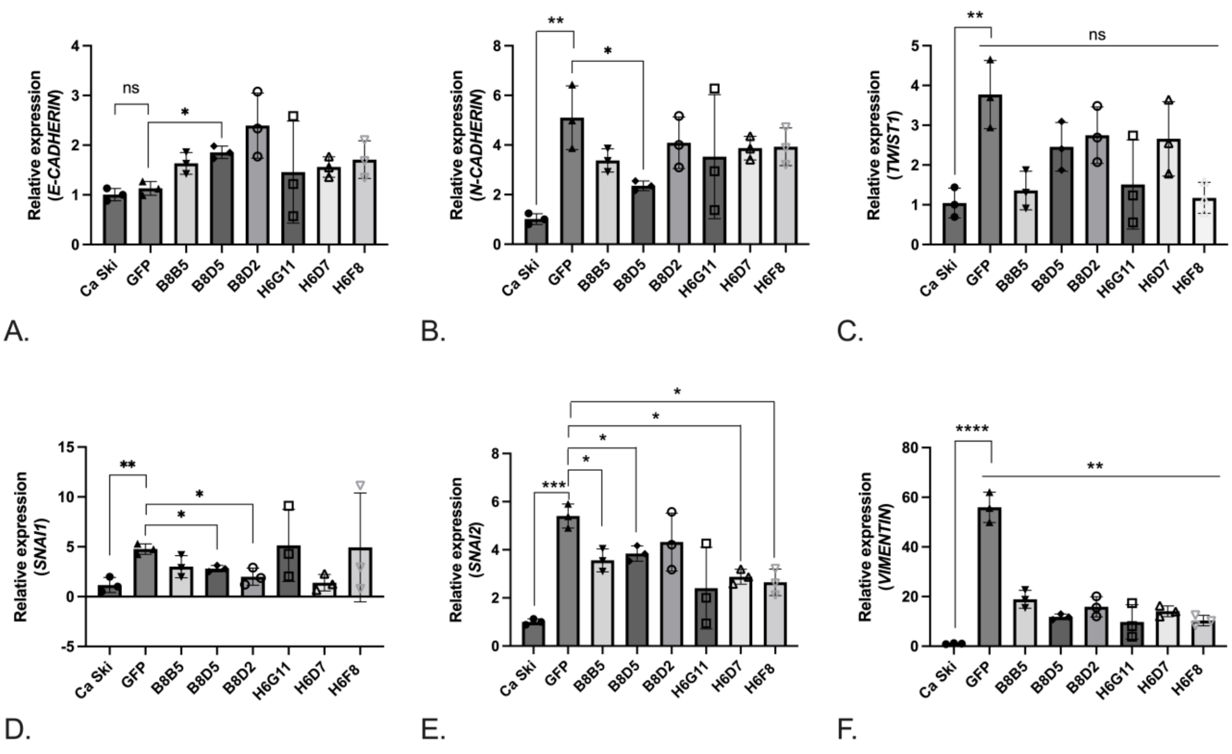

Supplementary Figure S7. Relative mRNA expression levels of *E-CADHERIN* (A), N-*CADHERIN* (B), *TWIST1* (C), *SNAI1* (D), *SNAI2* (E), *VIMENTIN* (F) in the derivatives of Ca Ski cells expressing variants of consensus HIV-1 FSU_A reverse transcriptase. Expression level was normalized to *GAPDH* expression and calculated as fold change compared to the parental Ca Ski line. Results are presented as mean ± SD. *Significant difference from the value of the migration rate in GFP subclone (∗p < 0.05; ∗∗p < 0.01; ∗∗∗p < 0.001; ∗∗∗∗p < 0.0001; Unpaired t test with Bonferroni correction).

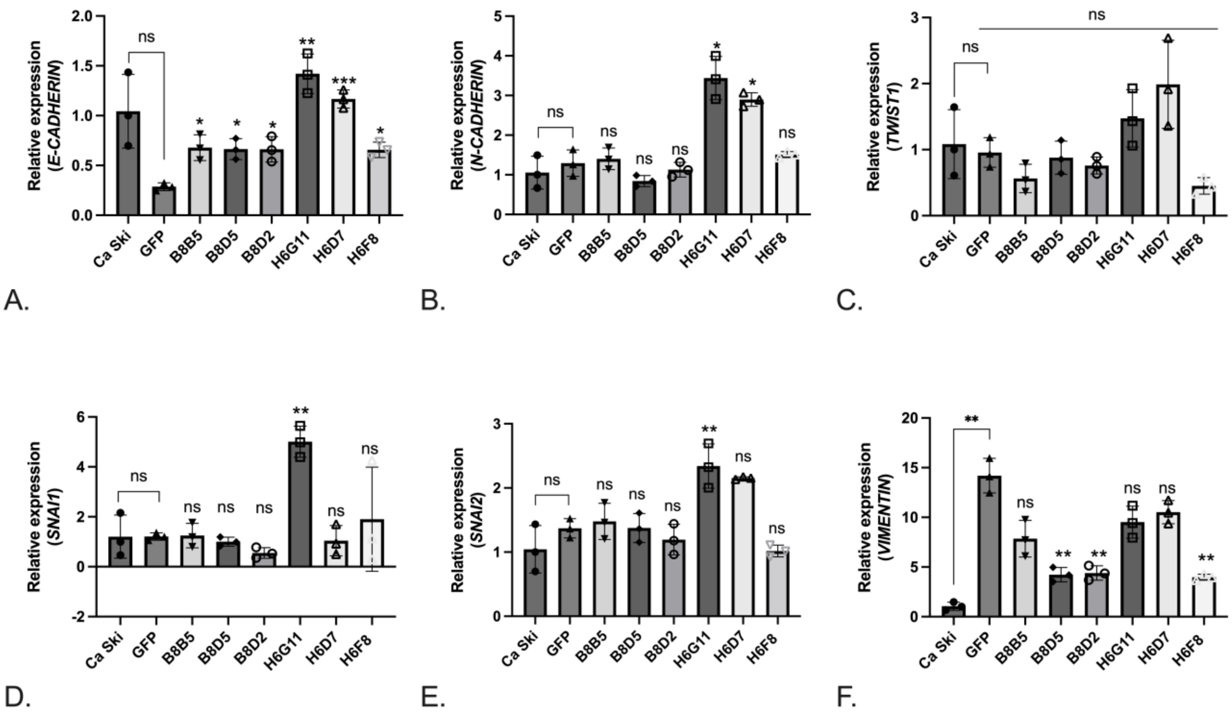

Supplementary Figure S8. Relative mRNA expression levels of *E-CADHERIN* (A), N-*CADHERIN* (B), *TWIST1* (C), *SNAI1* (D), *SNAI2* (E), *VIMENTIN* (F) in the derivatives of Ca Ski cells expressing variants of consensus HIV-1 FSU_A reverse transcriptase. Expression level was normalized to *GUSB* expression and calculated as fold change compared to the parental Ca Ski line. Results are presented as mean ± SD. *Significant difference from the value of the migration rate in GFP subclone (∗p < 0.05; ∗∗p < 0.01; ∗∗∗p < 0.001; ∗∗∗∗p < 0.0001; Unpaired t test with Bonferroni correction).

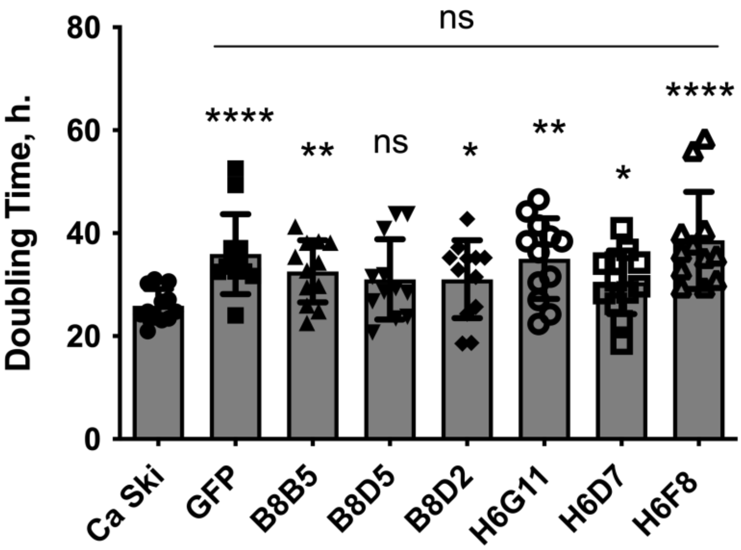

Supplementary Figure S9. The doubling time in the derivatives of Ca Ski cells expressing variants of consensus HIV-1 FSU_A reverse transcriptase over month of consecutive observations. *Significant difference from the value of doubling time in Ca Ski subclone (Kruskal - Wallis, n = 12, ∗p < 0.05; ∗∗p < 0.01; ∗∗∗p < 0.001; ∗∗∗∗p < 0.0001).

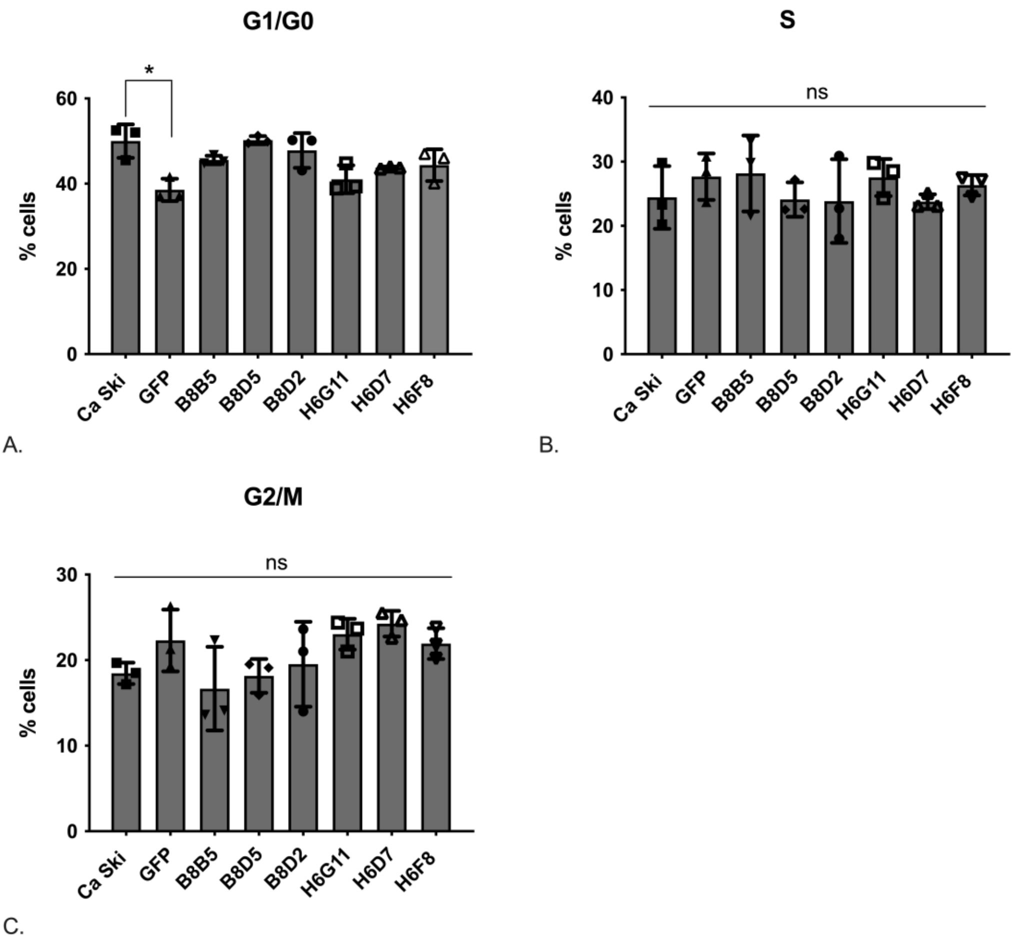

Supplementary Figure S10. Cell cycle distribution for subclones of Ca Ski expressing RT: G1/G0 phase (A), S phase (B), and G2/M phase (C). Distribution of cells in G1/G0, S, and G2/M areas was assessed using FlowJo software. Further analysis was performed automatically with preset G1 and G2 peaks and CVs at all samples. Data were analyzed using Kruskal-Wallis test with Dunn's multiple comparison test and in pairs using the Mann-Whitney test ($*p < 0.05$).

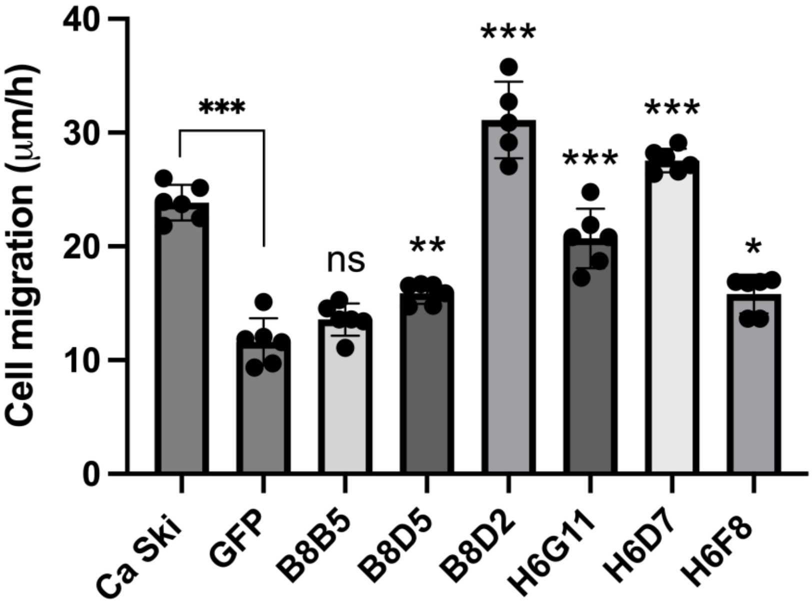

Supplementary Figure S11. Average migration rate of Ca Ski-derived subclones. Data are presented as mean ± SD (6 replicates). The previously described method (https://www.ncbi.nlm.nih.gov/pmc/articles/PMC5154238/) was used to estimate the obtained area values. For each individual repetition, the equation y=mx+b was built (by the points of the wound area at each individual hour), which made it possible to obtain the slope parameter (m). Then, U migration = |slope| / 2*L, where U is the speed of migration, slope is the slope (m), and L is the length of the wound. The slope of the resulting plots gives the migration rate in μm/h. The figure shows the migration rate for each iteration. Values are presented as mean ± SD from the assay run in triplicates. *Significant difference from the value of the migration rate in GFP subclone (∗p < 0.05; ∗∗p < 0.01; ∗∗∗p < 0.001; ∗∗∗∗p < 0.0001; Unpaired t test with Bonferroni correction).

Supplementary Table S2. Correlation of HIV-1 RT_A mRNA expression and protein production with phenotypic parameters: cell doubling time, distribution of cells by the phases of the cell cycle, and migration rate of Ca Ski and Ca Ski subclones (Spearman's rank correlation test).

	Doubling Time, h	WHA, migration rate, ?m/h	G1/G0, %	G2/M, %	S, %
RT_A mRNA, 2DCt	0,453463	0,292359	-0,161705	0,393087	0,047599
RT_A, fg/cell	0,300995	0,108265	-0,113500	0,324286	-0,027858

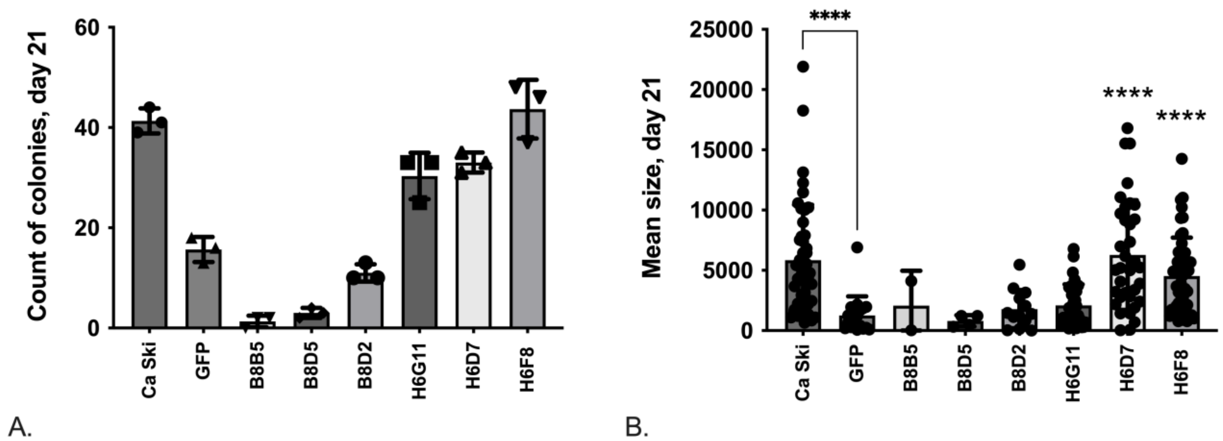

Supplementary Figure S12. Evaluation of clonogenic growth at days 21. Colony counts (A); The mean colony size (B). Statistical significance was assessed using the Kruskal-Wallis test with Dunn's multiple comparison test and in pairs using the Mann-Whitney test (∗p < 0.05; ∗∗p < 0.01; ∗∗∗p < 0.001; ∗∗∗∗p < 0.0001).
